# Comparison of Mobile Health Technology Use for Self-Tracking Between Older Adults and the General Adult Population in Canada: Cross-Sectional Survey

**DOI:** 10.2196/24718

**Published:** 2020-11-27

**Authors:** Mirou Jaana, Guy Paré

**Affiliations:** 1 Telfer School of Management University of Ottawa Ottawa, ON Canada; 2 Research Chair in Digital Health HEC Montreal Montreal, QC Canada

**Keywords:** mobile health, older adults, self-tracking, wearable technology, smart devices, mobile apps, survey, mobile phone, seniors, elderly

## Abstract

**Background:**

The burden of population aging and chronic conditions has been reported worldwide. Older adults, especially those with high needs, experience social isolation and have high rates of emergency visits and limited satisfaction with the care they receive. Mobile health (mHealth) technologies present opportunities to address these challenges. To date, limited information is available on Canadian older adults’ attitudes toward and use of mHealth technologies for self-tracking purposes—an area that is increasingly important and relevant during the COVID-19 era.

**Objective:**

This study presents contributions to an underresearched area on older adults and mHealth technology use. The aim of this study was to compare older adults’ use of mHealth technologies to that of the general adult population in Canada and to investigate the factors that affect their use.

**Methods:**

A cross-sectional survey on mHealth and digital self-tracking was conducted. A web-based questionnaire was administered to a national sample of 4109 Canadian residents who spoke either English or French. The survey instrument consisted of 3 sections assessing the following items: (1) demographic characteristics, health status, and comorbidities; (2) familiarity with and use of mHealth technologies (ie, mobile apps, consumer smart devices/wearables such as vital signs monitors, bathroom scales, fitness trackers, intelligent clothing); and (3) factors influencing the continued use of mHealth technologies.

**Results:**

Significant differences were observed between the older adults and the general adult population in the use of smart technologies and internet (*P*<.001). Approximately 47.4% (323/682) of the older adults in the community reported using smartphones and 49.8% (340/682) indicated using digital tablets. Only 19.6% (91/463) of the older adults using smartphones/digital tablets reported downloading mobile apps, and 12.3% (47/383) of the older adults who heard of smart devices/wearables indicated using them. The majority of the mobile apps downloaded by older adults was health-related; interestingly, their use was sustained over a longer period of time (*P*=.007) by the older adults compared to that by the general population. Approximately 62.7% (428/682) of the older adults reported tracking their health measures, but the majority did so manually. Older adults with one or more chronic conditions were mostly nontrackers (odds ratio 0.439 and 0.431 for traditional trackers and digital trackers, respectively). No significant differences were observed between the older adults and the general adult population with regard to satisfaction with mHealth technologies and their intention to continue using them.

**Conclusions:**

Leveraging mHealth technologies in partnership with health care providers and sharing of health/well-being data with health care professionals and family members remain very limited. A culture shift in the provision of care to older adults is deemed necessary to keep up with the development of mHealth technologies and the changing demographics and expectations of patients and their caregivers.

## Introduction

Population aging is a phenomenon that is associated with increased prevalence of chronic conditions worldwide [1-3]. In 2017, the global number of people 60 years and older was 962 million (including 137 million ≥80 years), and this number is expected to reach 2.1 billion by 2050 [3]. This growing population of older adults leads to an increased demand on the health systems for services, which are costly and require significant resources [4].

A recent Commonwealth survey of older adults in 11 countries investigated the challenges faced by adults aged ≥65 years at the social and health care levels [2,5]. The results showed that, across all surveyed countries, older adults, in general, and older adults with high needs, in particular (ie, multiple chronic conditions/functional challenges), experience social isolation and have high rates of emergency visits and general dissatisfaction with the quality of care they receive [2]. The current COVID-19 crisis has further catalyzed this problem. Older adults represent a group of the population that is at higher risk of death from severe acute respiratory syndrome associated with coronavirus, thus necessitating social distancing, which may lead to social isolation [6]. This confinement and social isolation can in turn have negative psychological effects and sleeping problems [7,8] and increased risk for early mortality [9]. In addition to potential social isolation, COVID-19 may have long-term effects on people with preexisting noncommunicable chronic diseases [10]. This is particularly observed with decrease in physical activity, unhealthy lifestyles during the COVID-19 crisis, and changes in the management of these conditions (eg, reduced outpatient visits, difficulty in diagnosing new conditions or recognizing deterioration in the existing ones) [10].

In Canada, wait times for various types of services (eg, doctors, specialists, emergency) have been historically longer compared to some other developed countries [11]. Generally, Canadians tend to be more frequent users of health services [11], and concerns have been growing about the ability of the public health care system to address the increasing needs of an aging population [12]. For instance, the population of Canadian adults aged 65 years and older reached 6.5 million in 2019 [13], and this number is expected to increase by 68% over the next 20 years [14]. This is particularly critical, given the high health care spending per capita on older adults and large use of services by this group [12].

Mobile health (mHealth) technologies present an opportunity to address the challenges associated with population aging and enable support for older adults in the community. mHealth refers to the use of mobile devices (eg, patient monitoring devices, mobile phones) to detect and monitor physiological changes and support medical and public health practice [15]. Prior research has examined the potential role of mHealth technologies in providing long-term support for older adults [16-18] and in monitoring chronic conditions often associated with older age [19-26]. Self-tracking devices in particular (eg, smart devices with mobile apps, fitness trackers, blood pressure monitors) have gained interest in recent years in light of their potential for monitoring and motivating individuals to remain healthy [27-31]. However, their use remains variable and less widespread among older adults [32], and prior research has reported risks associated with health information tracking, which may trigger negative emotions among patients with multiple chronic conditions and potential emotional draining in this group [33]. With the current COVID-19 crisis, calls for initiatives and efforts to bridge health information and communication technologies with the care for older adults have appeared in various countries as a preparedness mechanism and a mitigating approach against the current and future pandemics [7,10,34,35]. However, to date, limited information is available on older adults’ attitudes toward and their use of mHealth technologies for self-tracking purposes—an area that reveals to be increasingly important during and following this COVID-19 era.

This study, which is part of a larger program on digital health self-tracking [36], addresses this gap and presents the results of a national survey across all provinces in Canada, which assessed older adults’ familiarity with and use of mHealth technologies comprising mobile apps, smart devices, and wearables. Specifically, we report findings on the pattern of older adults’ use of mHealth technologies for self-tracking purposes and compare it to that of the general adult population. We also investigate the factors that influence the continued usage of mHealth technologies among older adults.

In order to address the objectives of this study, we propose a research model based on the work of Bhattacherjee [37] and Hong et al [38] and the expectation-confirmation theory [39]. In the present context, this model suggests that an older adult’s intention to continue using mHealth technologies is mainly influenced by his or her level of satisfaction, which is in turn affected by the extent to which his or her initial expectations toward mHealth technologies are confirmed, in addition to ease of use and perceived usefulness [40]. The latter factors also have direct links with usage continuance intention [38]. Hence, this study presents evidence on the extent to which older adults use mHealth technologies to self-track their health, compares their use of mHealth technologies to that of the general adult population, and analyzes the factors that influence the continued use of these technologies in the older population.

## Methods

### Study Design and Sampling

We present in this section the survey that was conducted in accordance with the Checklist* *for Reporting Results of Internet E-Surveys [41]. A web-based questionnaire was administered to a national sample of 4109 Canadian residents, aged 18 years or older, and who spoke English or French. The sample was selected from a proprietary web-based panel (AC Nielsen Company), which is one of the largest and most representative panels in Canada. To ensure representativeness of the overall population, the quota method was applied (age and gender) following a stratification by the geographic region. The ethics approval for the study was granted by the HEC Montréal’s research ethics committee. The older adult group in the sample consisted of all respondents aged 65 years and older and the general adult population in the sample consisted of respondents aged 18-64 years.

### Survey and Data Collection

The survey instrument consisted of 3 sections assessing the following items: (1) demographic characteristics, health status, and comorbidities; (2) familiarity with and use of mHealth technologies (ie, mobile apps, consumer smart devices/wearables such as vital signs monitors, bathroom scales, fitness trackers, intelligent clothing); and (3) factors influencing the continued use of mHealth technologies. The latter section also measured respondents’ satisfaction, ease of use, expectation confirmation, perceived usefulness, and intention to continue using mHealth technologies in the future.

Sociodemographic variables were measured using standardized indicators used in other international surveys [42-45]. These included gender, age, region, gross family income, education, occupation, and use of mobile phones and digital tablets. Health status was self-rated by respondents on a 5-point Likert scale (1=poor or fair, 5=very good or excellent), which is a common approach used in prior research [46]. A total of 11 chronic conditions were investigated (eg, diabetes, high blood pressure, cardiovascular disease, lung or respiratory cancer).

Respondents’ familiarity with mHealth technologies was assessed using a combination of items. A general question measured their familiarity with consumer wearables and smart medical devices on a 5-point Likert scale (1=not much at all familiar, 5=extremely familiar). Participants were also asked to indicate the devices that they owned by using descriptive terms that referred to 13 devices identified in the literature and available in the Canadian market. When participants indicated owning a specific device or wearable, they were asked to rate on a (1-7) scale (1=once a month or less, 7=many times a day) how often they used it in the past 3 months.

Three self-tracking profiles were identified in this study based on the respondents’ indication of their health tracking behavior. Those who regularly tracked one or more aspects of their health or well-being by using mHealth technologies, including mobile apps for health, consumer wearables (eg, fitness trackers), and smart medical devices (eg, blood pressure monitors), were defined as “digital trackers.” Respondents who regularly monitored one or more aspects of their health and well-being by using manual tools (ie, recording the information in writing) were defined as “traditional trackers.” All other respondents who did not regularly monitor any aspect of their personal health were considered as “nontrackers.”

The factors that are likely to influence the continued usage of mHealth technologies were captured. First, measures of perceived usefulness (7 items) and ease of use (4 items) were adapted from Davis [40] and rated on a 5-point Likert scale (1=strongly disagree, 5=strongly agree). We also adapted measures from Bhattacherjee [37] and Hong et al [38] to assess users’ satisfaction (3 items) and confirmation of initial expectations (3 items) on a 5-point Likert scale (1=strongly disagree, 5=strongly agree).

The survey instrument was first pretested during face-to-face interviews with 16 adults who were representative of the Canadian population in terms of gender, language, and age. Some small adjustments were made to the questionnaire following this step. A copy of the final survey instrument may be obtained from the authors upon request. Panel members were invited to participate in the study by email. Once participants clicked on the URL provided in the email letter, they were screened for the abovementioned eligibility criteria. All respondents read and approved an informed consent form prior to completing the questionnaire. Survey respondents were able to enter the survey at any point during the data collection period, ie, from January 11, 2017 to February 2, 2017. In accessing the web-based questionnaire, respondents were assigned a unique identifier and secret code (closed survey) that allowed them access to their data until the survey was done. Those who partly completed the survey were able to exit the questionnaire and return at a later time to enter additional data and to review and change their prior answers. Participants were rewarded gift cards (eg, Amazon, iTunes, Starbucks, magazine subscriptions) for survey completion. Rewards ranged in value from CAD $5 to CAD $75.

### Statistical Analysis

Data analysis was conducted to explore and better understand the pattern of use of these technologies and self-tracking behaviors by older adults in the community and compare it to that of the general adult population. Descriptive data analysis was performed to present an overview of the older adult group characteristics and their use of mHealth technologies. Bivariate analyses (two-sided *t* test for continuous variables and chi-square for categorical variables) were conducted to assess the differences between the 2 groups on these variables. Multinomial logistic regression tests were used to compare self-trackers (traditional and digital) and nontrackers, and Pearson correlation tests and partial least squares multiple regression analyses were used to analyze users’ appreciation of digital self-tracking devices. Data analyses were performed on SPSS Statistics v25 (IBM Corp) and SmartPLS 2.0 (SmartPLS GmbH).

## Results

### Sample Characteristics

Of the total study population of 4109 participants distributed across all provinces, 682 (16.6%) were aged 65 years and older (older adults) and 3427 (83.4%) were aged 18-64 years (general adult population), which represents the actual distribution of the older adults in the Canadian population [13]. Table 1 shows that a higher proportion of the older adults live on the east coast of Canada and British Columbia.

**Table 1:** Comparison of the characteristics of the older adults with those of the general adult population in this study.

Characteristics		Older adult population, n=682, n (%)	General population, n=3427, n (%)	Total, N=4109, n (%)	*P* value
**Gender**					<.001
	Male	400 (58.6)	1718 (50.1)	2118 (51.5)	
	Female	282 (41.3)	1709 (49.9)	1991 (48.5)	
**Region^a^**					<.001
	Atlantic provinces	56 (8.2)	237 (6.9)	293 (7.1)	
	Quebec	153 (22.4)	833 (24.3)	986 (24.0)	
	Ontario	265 (38.9)	1310 (38.2)	1575 (38.3)	
	Prairies	37 (5.4)	229 (6.7)	266 (6.5)	
	Alberta	50 (7.3)	387 (11.3)	437 (10.6)	
	British Columbia and territories	121 (17.7)	431 (12.6)	552 (13.4)	
**Highest education level^b,c^**					.09
	Primary and secondary school	181 (26.7)	758 (22.5)	939 (23.2)	
	College/CEGEP	177 (26.1)	972 (28.8)	1149 (28.4)	
	University undergraduate	207 (30.6)	1093 (32.4)	1300 (32.1)	
	University graduate	112 (16.5)	549 (16.3)	660 (16.3)	
**Employment**					<.001
	Full-time	37 (5.4)	1921 (56.1)	1958 (47.6)	
	Part-time	44 (6.4)	385 (11.2)	429 (10.4)	
	Retired	587 (86.1)	350 (10.2)	937 (22.8)	
	Other	14 (2.1)	771 (22.5)	785 (19.1)	
**Income^d^**					<.001
	<$20,000	32 (5.7)	236 (8.1)	268 (7.7)	
	$20,000-$39,999	123 (22.1)	461 (15.7)	584 (16.7)	
	$40,000-$59,999	131 (23.5)	482 (16.4)	613 (17.6)	
	$60,000-$79,999	95 (17.1)	465 (15.9)	560 (16.1)	
	$80,000-$99,000	74 (13.2)	424 (14.4)	498 (14.3)	
	≥$100,000	102 (18.3)	863 (29.4)	965 (27.7)	
**Chronic conditions^e^**					<.001
	Yes	342 (51.4)	939 (28.0)	1281 (31.9)	
	No	323 (48.6)	2413 (72.0)	2736 (68.1)	
**Current health status^b^**					.87
	Very poor/poor	63 (9.2)	339 (9.9)	402 (9.8)	
	Good	345 (50.7)	1724 (50.3)	2070 (50.4)	
	Very good/excellent	274 (40.2)	1364 (39.8)	1638 (39.9)	
**Tracking health measures**					<.001
	Manual self-tracking	307 (45.0)	744 (21.7)	1051 (25.6)	
	Electronic self-tracking	121 (17.8)	1547 (45.1)	1668 (40.6)	
	No self-tracking	254 (37.2)	1135 (33.1)	1389 (33.8)	

^a^Atlantic provinces include Nova Scotia, New Brunswick, New Foundland and Labrador, and Prince Edward Island; Prairies include Manitoba and Saskatchewan; Territories include Nunavut, Yukon, and Northwest territories.

^b^Significant differences were observed between seniors and the general population on all variables except for “Current health status” and the “Highest level of education”.

^c^There were 5 and 55 nonrespondent in the older adult group and the general adult population, respectively.

^d^There were 125 and 496 nonrespondents in the older adult group and the general adult population, respectively. All income data are provided in Canadian dollars (CAD $1=US $1.31).

^e^There were 17 and 75 nonrespondents in the older adult group and the general adult population, respectively.

When comparing the older adult population with the general adult population, significant differences were observed in all the characteristics, with the exception of education level and reported health status; comparable educational levels were noted in the 2 groups and the perceived health status was reported as good-to-excellent in both groups. Compared to the general adult population, the older adult population had a larger number of men, who were retired, and had an annual income below CAD $60K. Of the 682 older adults, 342 (50.1%) indicated having one or more chronic conditions compared to 939 (27.4%) respondents of the total general adult population of 3427. Among the 62.8% (428/682) of the older adults who reported self-tracking of their health, 17.7% (121/682) did so electronically (digital trackers) compared to 45.1% (1547/3426) in the general adult population. The majority of the older adults reported tracking their health parameters manually (traditional trackers).

### Internet and Smart Technologies

Table 2 shows significant differences between the older adult population and the general adult population in terms of internet and smart technology use. Of the 682 older adults, 323 (47.3%) and 340 (49.8%) reported using a smartphone and a digital tablet, respectively, as compared to 2887 (84.2%) and 2337 (68.2%) respondents of the 3427 respondents in the general adult population. Among the 463 older adults using smartphones/digital tablets (out of 682 participating in this study), only 91 respondents (19.6%) downloaded ≥1 mobile apps and 314 (67.8%) indicated accessing the internet on a daily basis versus 45.6% (1406/3082) and 87.9% (2709/3082) in the general adult population, respectively. When asked about their familiarity with smart devices/wearables for health, 82.7% of the older respondents (383/463) indicated having heard of these technologies, but only 32.1% of the older adults (123/383) were somewhat familiar or very familiar with them.

**Table 2:** Comparison of the internet and mobile health technology use of the older adults with that of the general adult population.

Use of internet and mobile health technology		Older population	General population	Total	*P* value
**Using a smartphone**		682	3427	4109	<.001
	Yes, n (%)	323 (47.4)	2887 (84.2)	3210 (78.1)	
	No, n (%)	359 (52.6)	540 (15.8)	899 (21.9)	
**Using a digital tablet**		682	3426	4109	<.001
	Yes, n (%)	340 (49.9)	1997 (58.3)	2337 (56.9)	
	No, n (%)	342 (50.1)	1429 (41.7)	1772 (43.1)	
**Accessing internet using smartphone/digital tablet^a^**		463	3082	3545	<.001
	Never, n (%)	52 (11.2)	89 (2.9)	141 (4.0)	
	Less than daily, n (%)	97 (21.0)	284 (9.2)	381 (10.7)	
	Daily, n (%)	314 (67.8)	2709 (87.9)	3023 (85.3)	
**Downloaded ≥1 mobile apps on smartphone/digital tablet^a^**		463	3082	3545	<.001
	Yes, n (%)	91 (19.6)	1406 (45.6)	1497 (42.2)	
	No, n (%)	372 (80.3)	1676 (54.4)	2048 (57.8)	
**Heard of smart devices/wearables for health**		463	3082	3545	.03
	Yes, n (%)	383 (82.7)	2667 (86.5)	3050 (86.0)	
	No, n (%)	80 (17.3)	415 (13.5)	495 (14.0)	
**Familiarity with smart devices/wearables for health^a^**		383	2667	3050	<.001
	Slightly familiar, n (%)	260 (67.9)	1227 (46.0)	1487 (48.7)	
	Somewhat familiar, n (%)	103 (26.9)	973 (36.5)	1076 (35.3)	
	Very familiar, n (%)	20 (5.2)	467 (17.5)	487 (16.0)	

^a^The total values in the rows indicate the number of respondents for that category, which may be lower than the total number of older adults and the general adult population.

### Mobile Apps for Health and Well-being

Table 3 compares the use of mobile apps for health/well-being between the older adult population and the general adult population. Among the 91 older adults who downloaded ≥1 mobile apps (presented in Table 2), 78 respondents (86%) indicated having used mobile apps for health/well-being in the last 3 months, which is comparable to that of the general population (1257/1406 respondents, ie, 89.4%). No significant differences were noted in relation to the number of mobile apps for health used nor in the extent of data sharing between the 2 groups. Among the 30 older adults that reported sharing data, 21 respondents (70%) indicated sharing data with family members, and 4 respondents (13%) reported sharing data with their friends and doctors. Interestingly, 38% of the older adults (29/77) reported using these mobile apps for 1-2 years as compared to 22.1% in the general population (269/1219). However, it is important to note that no significant differences were observed between the older adults and the general population who used mobile apps for health in the factors that affect their use (ie, perceived ease of use, perceived usefulness, and expectation confirmation). The overall satisfaction and intention to continue using mobile apps were favorable in both groups.

**Table 3:** Comparison of the use and perceptions of mobile apps for health between older adults who indicated downloading these apps and their counterparts in the general adult population.

Use and perceptions		Older population	General population	Total	*P* value
**Mobiles apps for health/well-being used (last 3 months)^a^**		78	1257	1335	.06
	1 app, n (%)	40 (51)	514 (40.9)	554 (41.5)	
	2 apps, n (%)	26 (33)	406 (32.3)	432 (32.4)	
	≥3 apps, n (%)	12 (15)	337 (26.8)	349 (26.1)	
**Duration of use of mobile health/well-being apps^a^**		77	1219	1296	.007
	<1 year, n (%)	39 (51)	790 (64.8)	829 (64.0)	
	1-2 years, n (%)	29 (38)	269 (22.1)	298 (23.0)	
	>2 years, n (%)	9 (12)	160 (13.1)	169 (13.0)	
**Sharing of health/well-being data from apps^a^**		77	1238	1315	.50
	Yes, n (%)	30 (39)	436 (35.2)	466 (35.4)	
	No, n (%)	47 (61)	802 (64.8)	849 (64.6)	
Satisfaction with mobile apps, mean (min-max)^b^		3.79 (1.67-5)	3.78 (1-5)	3.70 (1-5)	.89
Ease of use, mean (min-max)^b^		4.00 (1.5-5)	3.95 (1-5)	3.95 (1-5)	.55
Expectation confirmation, mean (min-max)^b^		3.74 (1.67-5)	3.60 (1-5)	3.61 (1-5)	.12
Perceived usefulness, mean (min-max)^b^		3.59 (1.25-5)	3.56 (1-5)	3.56 (1-5)	.78
Intention to continue using mobile apps, mean (min-max)^b^		3.97 (1-5)	3.91 (1-5)	3.92 (1-5)	.61

^a^The total values in the rows indicate the number of respondents for that category, which may be lower than the total number of older adults and the general adult population.

^b^The means represent the average of 4 questions that constitute each scale (satisfaction with mobile apps, ease of use, expectation confirmation, perceived usefulness, and intention to continue using mobile apps). Continuous variables were measured on a 5-point Likert scale.

### Smart Devices/Wearables for Health

Among the 383 older adults in the sample who had heard of smart devices/wearables (as presented in Table 2), 47 respondents (12.2%) reported having ≥1 smart devices and indicated currently using them, while another 35 respondents (9.1%) reported having these devices but not using them; the remaining 78.6% (302/383) indicated not having smart devices/wearables. The majority of the older adults had only 1 device as opposed to the general adult population with more respondents indicating having 2 or more devices (see Table 4), and the most common type of smart devices/wearables used was bracelet/wristband watches.

**Table 4:** Comparison of the use of smart devices/wearables for health between older adults who own these devices and their counterparts in the general adult population.

Use and perceptions		Older population	General population	Total	*P* value
**Having ≥1 smart device/wearables for health, n (%)^a^**		384	2667	3051	<.001
	Yes, and use them	47 (12.2)	533 (20.0)	580 (19.0)	
	Yes, and stopped using them	24 (6.3)	236 (8.9)	260 (8.5)	
	Yes, and never used them	11 (2.8)	164 (6.1)	175 (5.7)	
	No	302 (78.6)	1734 (65.0)	2036 (66.7)	
**Number of smart devices or wearables owned, n (%)^a^**		47	531	578	.049
	1	39 (83)	368 (69.3)	407 (70.4)	
	≥2	8 (17)	163 (30.7)	171 (29.6)	
**Duration of use of smart devices/wearables, n (%)** ^a^		45	530	575	.19
	<1 year	19 (42)	297 (56.0)	316 (55.0)	
	1-2 years	18 (40)	153 (28.9)	171 (29.7)	
	>2 years	8 (18)	80 (15.1)	88 (15.3)	
**Use of smart devices/wearables in partnership with health care provider, n (%)^a^**		46	533	579	.17
	Yes	3 (7)	73 (13.7)	76 (13.1)	
	No	43 (94)	460 (86.3)	503 (86.9)	
Satisfaction with smart devices/wearables, mean (min-max)^b^		4.08 (2-5)	4.07 (1-5)	4.08 (1-5)	.98
Ease of use, mean (min-max)^b^		4.20 (2-5)	4.21 (1-5)	4.21 (1-5)	.92
Expectation confirmation, mean (min-max)^b^		3.78 (1.67-5)	3.89 (1-5)	3.88 (1-5)	.31
Perceived usefulness, mean (min-max)^b^		3.66 (1.50-5)	3.82 (1-5)	3.80 (1-5)	.15
Intention to continue using smart devices/wearables, mean (min-max)^b^		4.22 (1-5)	4.26 (1-5)	4.25 (1-5)	.75

^a^The total values in the rows indicate the number of respondents for that category, which may be lower than the total number of older adults and the general adult population.

^b^The means represent the average of 4 questions that constitute each scale (satisfaction with mobile apps, ease of use, expectation confirmation, perceived usefulness, and intention to continue using mobile apps). Continuous variables were measured on a 5-point Likert scale.

When asked about the types of devices used, the answers also varied between the 2 groups. The most commonly reported devices were bracelets/wristbands. The adults in both the groups who reported using smart devices/wearables did not differ significantly in relation to the duration of use of these technologies and the extent of use in partnership with a health care provider, which was relatively low among the respondents, that is, 7% of the older adults (3/46) and 13.7% (73/530) of the general adult population. As in the case of mobile app use for health, no significant differences were observed in the factors that affect the use of smart devices/wearables (ie, perceived ease of use, perceived usefulness, and expectation confirmation) between the older adult and the general adult population. The overall satisfaction with and the intention to continue using these smart devices/wearables were high. The older adults who used wearables and smart devices reported being very satisfied (mean 4.1 on the 5-point Likert scale), perceived their devices to be user-friendly (mean 4.2), and had a firm intention to continue using them in the future (mean 4.2). Importantly, respondents perceived these devices as relatively useful. About 6 out of 10 users said that they maintained or improved their health status by using digital self-tracking devices. Approximately 66% (31/47) of the older adult users of smart devices/wearables reported they were more informed or more knowledgeable about their health condition due to the use of these devices. For their part, 53% (25/47) of the older adult users said they felt more confident taking care of their health or more autonomous in the management of their condition. Interestingly, feeling less anxious about one’s own health and having more informed discussions with a doctor were not perceived as major benefits among the older adult group.

### Perception of Smart Devices and Self-tracking Behaviors Among Older Adults

Cronbach alpha was used to assess the reliability of the measures related to satisfaction and use of mHealth technologies, which were included in this study. The results (see Table 5) show that all the measures exceed the .70 threshold of statistical significance [47]. The validity of the variables was also supported; the square root of the variance shared by each variable and its respective items (diagonal) was greater than the intercorrelations between the variables.

**Table 5:** Variance shared by the variables considered in this study.^a^

Variables	Number of items	Cronbach α	Perceived usefulness	Perceived ease of use	Confirmation of expectations	User satisfaction	Intention to continue usage
Perceived usefulness	4	.86	*0.82*	0.42^b^	0.79^b^	0.70^b^	0.71^b^
Perceived ease of use	4	.88		*0.84*	0.65^b^	0.62^b^	0.45^b^
Confirmation of expectations	3	.70			*0.83*	0.78^b^	0.63^b^
User satisfaction	3	.88				*0.89*	0.74^b^
Intention to continue usage	3	.93					*0.90*

^a^The diagonal (italicized values in the table) refers to the square root of the variance shared by each variable and its respective items. The values off the diagonal refer to the intercorrelations between the variables. The values in the lower part of the table are a mirror of those in the upper part above the diagonal.

^b^The correlation was significant at *P*<.01.

**Figure 1:**
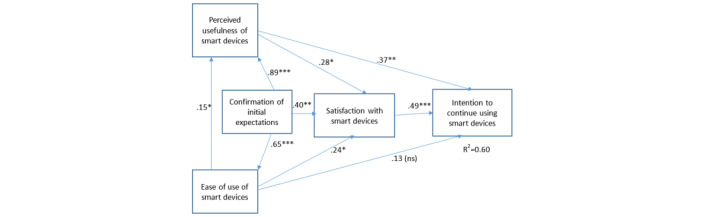
Results of the partial least squares regression analyses that were performed to test the associations between satisfaction, initial expectations, and intention to continue using smart devices/wearables. * *P*<.05; **P<.01; ***P<.005; ns: nonsignificant.

Partial least squares regression analyses that were performed to test the associations between satisfaction, initial expectations, and intention to continue using smart devices/wearables (Figure 1) showed that all relationships but one were supported, and the model explained 60% of the variance in the dependent variable. These results indicate that expectation confirmation is strongly related to ease of use, perceived usefulness, and user satisfaction, which in turn affect the older adults’ intentions to continue using these mHealth technologies.

Last, a multinomial logistic regression, including sociodemographic and health status variables, was performed to calculate the odds ratios describing the odds of tracking one’s own health using traditional or digital devices compared with the odds of nontracking (reference category) among the older adult group. The traditional .05 criterion of statistical significance was employed for all tests. Addition of the predictors to a model that contained only the intercept significantly improved the fit between model and data; *χ*^2^_34_(n=682)=49.46, Nagelkerke *R*^2^=0.11, *P*<.01.

As indicated in Table 6, our analyses showed no statistically significant differences between the groups (traditional trackers, digital trackers, and nontrackers) in terms of gender, education level, occupation, and perceived health condition. However, significant differences were observed in terms of region (*P*=.005 and *P*=.03 for traditional trackers and digital trackers, respectively, in Alberta) and chronic conditions (*P*<.001 and *P*=.003 for traditional trackers and digital trackers, respectively). Older adults living in the province of Alberta were 4.9 times more likely to be in the digital self-tracking group than the older adults living in other Canadian regions. Compared with older adults living with no chronic condition, older adults with chronic conditions were 0.4 times less likely to be digital self-trackers.

**Table 6:** Multinomial logistic regression results^a^.

Characteristics		Traditional trackers, n=307		Digital trackers, n=121	
		OR^b^ (95% CI)	*P* value	OR^b^ (95% CI)	*P* value
Intercept		N/A^c^	<.001	N/A	<.001
**Gender**					
	Female	1.176 (0.761-1.817)	.47	1.144 (0.656-1.996)	.63
**Region**					
	Atlantic provinces	1.342 (0.582-3.094)	.49	1.098 (0.379-3.183)	.86
	Quebec	1.896 (0.983-3.658)	.06	1.322 (0.585-2.988)	.50
	Ontario	1.503 (0.828-2.729)	.18	1.001 (0.478-2.096)	>.99
	Prairies	1.270 (0.487-3.309)	.62	0.793 (0.220-2.861)	.72
	Alberta	6.053 (1.719-21.312)	.005	4.914 (1.221-19.775)	.03
**Education**					
	Primary and secondary school	1.064 (0.539-2.102)	.66	0.623 (0.274-1.616)	.37
	College/CEGEP	1.371 (0.686-2.738)	.37	0.623 (0.541-2.960)	.59
	University undergraduate	1.270 (0.675-2.392)	.46	0.832 (0.571-2.675)	.59
**Occupation**					
	Full-time employment	1.078 (0.181-6.422)	.93	0.633 (0.099-4.438)	.67
	Part-time employment	1.972 (0.342-11.375)	.45	0.799 (0.118-5.404)	.82
	Retired	1.596 (0.333-7.649)	.56	0.699 (0.139-3.525)	.66
**Perceived health condition**					
	Very poor/ poor	0.524 (0.240-1.14)	.10	0.734 (0.275-1.955)	.54
	Fair or good	1.094 (0.692-1.730	.70	1.249 (0.697-2.240)	.45
≥1 chronic disease(s)		0.439 (0.281-0.686)	<.001	0.431 (0.245-0.758)	.003

^a^Reference category: nontrackers (n=254).

^b^OR: odds ratio.

^c^N/A: not applicable.

## Discussion

### Study Relevance

This study investigates older adults’ use of mHealth technologies in comparison to that of the general adult population and assesses the pattern of use of these technologies for self-tracking purposes. The surveyed older adult population differed significantly from the general population in relation to the sociodemographic variables. This stresses the importance of having a closer examination of older adults’ use of mHealth technologies for self-tracking purposes separately from the general adult population, which would inform future research, practice, and policy efforts in this area.

### Principal Findings

Although there were significant differences between older adults and the general adult population in the use of internet and smart technology, a considerable number of older adults reported using them. Specifically, 47.3% (323/682) and 49.8% (340/682) of the older population (65 years and older) reported using a smartphone or a digital tablet, respectively, and 67.8% (314/463) indicated accessing the internet on a daily basis. A large number of Canadian older adults in the community have already acquired these technologies, which presents an opportunity to leverage them beyond basic communication use to support their well-being by enhancing social connectedness and improving the management of their health conditions [48,49].

Despite the comparable good-to-excellent health status reported by older adults and the general adult population, the prevalence of chronic conditions was significantly higher among the older adult group compared to that among the general adult population (*P*<.001), which necessitates close monitoring and management of their health and conditions. Therefore, it is important to leverage existing technologies that can support their health and well-being needs in the community and potentially connect them with caregivers and health care providers. This is particularly relevant in relation to wearables (eg, wristbands, pedometers) and mobile apps that allow users to store and monitor health-related data. Prior research has discussed the important role of technology to support the ability of older adults to remain at home, improve their quality of life and health outcomes, and enhance family caregivers’ and health care professionals’ access to relevant information [16,50]. This is in line with the findings of this study that showed a high satisfaction rate with mHealth technologies and favorable conditions for their use.

Nevertheless, this study demonstrates that the potential of mHealth technologies for self-tracking purposes has not been fully captured yet in the context of older adults. Although 62.8% (428/682) of the older adults reported tracking their health measures, the majority did so manually, which may compromise the process, given the risk of losing information and the difficulty in sharing it with health care providers and caregivers. This considerable number of older adults tracking their health measures is indicative of the need and interest among this group to monitor their health. In the absence of the widespread use of personal health records, older adults do not have options for tracking and monitoring their health status but through their own initiative; as such, many seem to resort to the traditional manual recording of their health. This may be an indication of limited knowledge that they may have on mHealth technologies and how they work or a lack of funding and incentives to acquire and use these technologies (ie, from the health care providers, government, and caregivers). Surprisingly though, older adults with one or more chronic conditions appeared to be mostly nontrackers, which raises concerns as to the extent to which mHealth technologies are indeed benefitting the older adults most in need of them.

In light of these findings, it is critical to develop strategies to enhance older adults’ awareness and knowledge of the existing mHealth technologies available at their disposal and how to use them and encourage family physicians and allied health professionals to communicate about these options with them. In addition, it is equally important to understand older adults’ priorities and self-tracking needs in order to offer technologies suitable to address these needs [32]. This is particularly relevant in light of recent studies in other countries showing that older adults’ acceptance of mobile apps can be improved by informing them about the potential benefits of these technologies [51] and that older adults agree to share collected data through in-home monitoring and sensors with professional caregivers and demand participation in decisions about technology [52].

Interestingly, the majority of the mobile apps downloaded by the surveyed older adults consisted of apps used for health and well-being, reflecting a “targeted” use of these technologies by older adults. Around half of the older adults who reported mobile app use in the past 3 months specified using 2 or more of these apps. This is indicative of the perceived benefits of these technologies by older adults and can also reveal a level of comfort and interest in the use of these mobile apps over time. Once older adults start using mobile apps for health, their interest and willingness to use more than one mobile app over a long period of time was confirmed (Table 3 shows higher proportion of older adults reporting >1-year use compared to the general population). Future studies should investigate the motivating factors that facilitate their embracement of mHealth technologies to develop strategies that would enable a broader range of older adults to benefit from them.

It is also important to note that a low proportion of respondents among the older adults and in the general population, that is, 39% (30/77) and 35.2% (436/1238), respectively, indicated either sharing data from mobile apps or using smart devices/wearables in partnership with health care providers. Hence, it appears to be a disconnect between the actual needs and willingness of the older adults in the community to use mHealth technologies and the ability and readiness of health care providers to leverage these tools to support the care provided for these individuals. Despite previous efforts to explore the factors that affect health information technology adoption by older adults in the community [26], we have very limited information about the facilitators and barriers that play a role in bridging this disconnect and enabling more optimal use of mHealth technologies for older adults’ care.

The partial least squares regression analyses confirmed that expectation confirmation is strongly related to ease of use, perceived usefulness, and user satisfaction. Hence, it is critical to adequately manage older adults’ initial expectations to ensure greater adherence and continued usage of wearables and smart devices. These initial expectations may be considered as the anchor for the subsequent behavior of older adults, and their acceptance and the use of these technologies, and which may be shaped by the environment in which they live. Caregivers and family members, peers, as well as health care providers can play a significant role in shaping these initial expectations and the subsequent benefits that older adults may reap out of using these technologies. Interestingly, the results of this study show that older adults living in Alberta were 4.9 times more likely to be in the digital self-tracking group compared to older adults in other regions. Alberta is a province known to attract young families and is known for its highest rate of workforce growth. This may have implications for older adults living in this province who are surrounded by a younger population heavily immersed in technology and who may have expectations in relation to the role of mHealth technologies in the care for their older persons.

A culture shift in the provision of care to Canadian older adults living in the community is deemed necessary in order to keep up with the development of mHealth technologies and the changing demographics and expectations of patients and their caregivers. This is particularly important in light of the results in this study that show that older adults living with chronic conditions are 0.4 times less likely to be digital self-trackers. This is a “missed opportunity” at the community level as the individuals who may benefit most from mHealth technologies (ie, older adults with chronic conditions) do not seem to be actually using them. Given this state, how can we make this leap and paradigm shift? Evidently, this shift cannot come along without paralleled changes at the health system level in relation to existing policies, reimbursement modalities, and the structure of health care services delivery. In order to optimize the use of mHealth technologies to support older persons in the community, who need and are capable of using them, it is important that health care providers integrate data gathered through these smart devices in the delivery of care to them.

With the recent COVID-19 crisis, we have seen a rapid uptake of virtual care worldwide, which has been catalyzed by a dire need to provide “remote care” to a vulnerable population (ie, older adults) and a facilitated reimbursement approach. For example, as of May 1, 2020, the Ministry of Health and Long-Term Care in the province of Ontario, one of the largest health jurisdictions in Canada, implemented new temporary fee schedule codes that cover virtual assessments and provision of services [53]. Despite this agile adaptation during the time of crisis, it is equally important to develop long-term plans to leverage technologies to support the care for Canadian older adults, which may require reforms at the health system level.

Before the COVID-19 crisis, we had started to witness unconventional changes in this area in some Canadian provinces with initiatives that allowed patients to leverage wearables and smart devices to support their health. Alberta, for example, had released a personal health record initiative allowing patients to collect and store their own health data by using wearables and smart medical devices and manage authorizations for accessing these data. Other provinces, including Quebec, Nova Scotia, and Saskatchewan, are following this lead with health information portals giving patients more access and control over their health data. These initiatives are promising; however, they have to be paralleled and supported by changes at the policy and reimbursement levels to close the loop and encourage health care providers to endorse new technologies as integrated components in the delivery of health services for older adults and enablers for improved quality of care.

It is worth noting that the consistent high satisfaction of older adults with mobile apps and smart devices/wearables and their intention to continue using them is a positive indication of the evolving expectations of the older adult population and a potential game changer for the future of care for older adults. The results of this study confirm that once mobile apps and smart devices/wearables are used, the perceived ease of use and usefulness of these technologies do not vary by age of the users. As the older adult population continues to grow to include people currently still in the workforce and using technology in their daily lives (eg, mobile apps, smart devices), the demand for more connectedness with health care providers and better response from the health care system in a networked society will likely increase.

### Limitations and Future Research

This study presents contributions to an underresearched area on older adults and mHealth technology use. These findings are the first step toward understanding the behaviors and attitudes of older adults toward these technologies. By unveiling the actual prevalence of mHealth technology use among the Canadian older adult population and exploring their familiarity and satisfaction with these technologies, we set the stage for future research to investigate the optimal environment and predictors for their effective use [54].

At present, there are still significant differences between older adults and the general adult population in relation to the use of mHealth technologies. This necessitates a particular focus on older adults in future studies in order to better understand the needs and perceived facilitators and barriers for the use of these technologies this group. However, interestingly, both groups considered in this study demonstrated similarities in terms of limited current use of mobile apps and wearable devices for sharing data in partnership with health care providers. This calls for future research, which extends to the whole population, to better understand the underlying reasons and challenges in this area and study the feasibility and readiness of health care providers to leverage these tools to support the care that they provided to their patients.

Last, it is important to note some limitations associated with the study design and breadth of data. The data set used in this study is from a single country, thereby limiting the generalizability of the findings. In addition, the web-based survey was completed by respondents who had access to the internet, which may preclude representativeness of potential respondents with no internet access. Given the cross-sectional nature of the survey, a full assessment of the predictors of older adults’ use of mHealth technologies as well as an evaluation of the variation in their behaviors over time, especially in relation to changes in their health conditions, was not feasible. Furthermore, given the exploratory nature of the study and the focus on mobile apps and smart devices/wearables, limited data were collected on the functional ability of the older adults, their level of independence and health condition, and other sociodemographic characteristics that may play a role in shaping their use of these technologies. Future studies should take these factors into account to better understand the variation in the use of mHealth technologies by older adults in the community and determine the optimal conditions in which these technologies can best benefit them.

### Conclusion

The burden of population aging and the associated chronic conditions is observed worldwide. Mobile technologies present an opportunity to address the challenges faced by older adults in relation to their health and the care that they receive. This study shows that a considerable number of older adults are familiar with and use these technologies. Importantly, older adults who use mHealth technologies are highly satisfied with them and plan to continue using them in the future. Understanding why older adults who are familiar with mHealth technologies are not using them would inform progress in this area. In particular, leveraging these mHealth technologies for older adults who need and may benefit from them, in partnership with family physicians and allied health care professionals remains very limited at present. The current development and deployment of various personal health record initiatives in Canada appear as a promising avenue to facilitate bidirectional health information exchanges between health care providers and patients, including older adults.

## Abbreviations

mHealth: mobile health
